# Photoreceptors as central hubs of developmental plasticity and environmental adaptation

**DOI:** 10.1016/j.mocell.2026.100390

**Published:** 2026-08-19

**Authors:** Hyeona Hwang, Soeun Han, Hyunwoo Cho

**Affiliations:** Department of Industrial Plant Science and Technology, Chungbuk National University, Cheongju 28644, Korea

**Keywords:** Environmental response, Photoreceptors, Plant phytochrome, Vascular development

## Abstract

Plant photoreceptors are well known for their roles in light-dependent developmental processes such as photomorphogenesis, shade avoidance, and circadian regulation. These canonical functions involve light perception through specific receptors, including phytochromes and cryptochromes, and downstream transcriptional reprogramming mainly in the context of early seedling growth. Beyond their classical roles, recent studies have revealed that photoreceptors also act as integrators of diverse environmental signals, contributing to abiotic stress adaptation, root development, and vascular differentiation. Notably, these non-canonical functions involve direct protein–protein interactions, post-translational modifications, and hormonal crosstalk, enabling photoreceptors to respond to environmental cues beyond light. Moreover, photoreceptor activity has been detected in tissues with limited or no light exposure, such as roots and adult vascular tissues, where they contribute to developmental and stress-responsive processes. In selected cases, particularly for phyB, non-light stress-related signals have been reported to modulate photoreceptor stability or subnuclear organization, suggesting that the functional repertoire of photoreceptors may extend beyond traditional photoperception. This review summarizes emerging insights into the non-canonical functions of photoreceptors in abiotic stress adaptation and root and vascular development, with particular focus on light-independent signaling, root-local photoreceptor activity, and non-cell autonomous light signaling. By integrating recent findings, we aim to provide a broader perspective on how photoreceptors serve as central regulators of plant plasticity in response to both light and non-light environmental factors, spanning from early to adult developmental programs.

## Graphical Abstract

**Figure:**
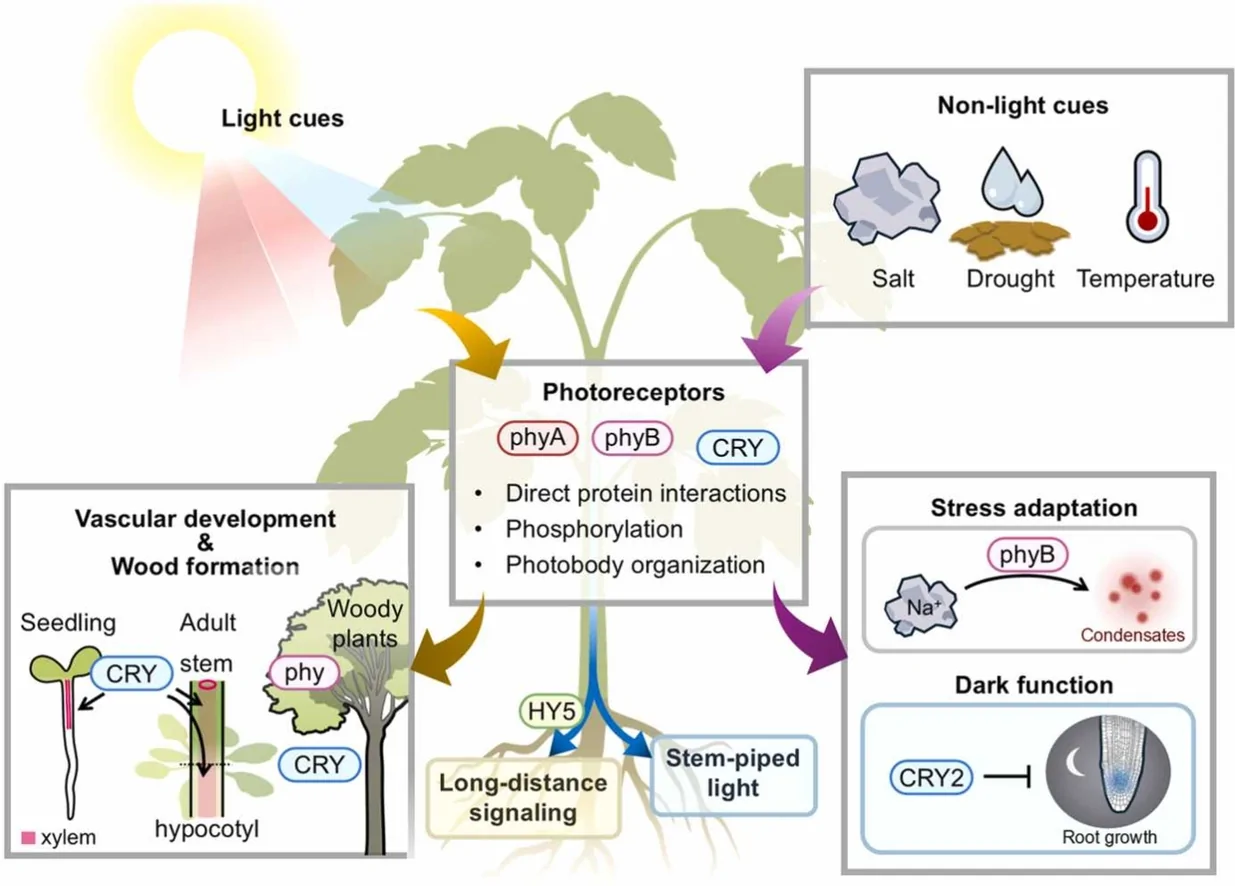

## INTRODUCTION

**Fig. 1:**
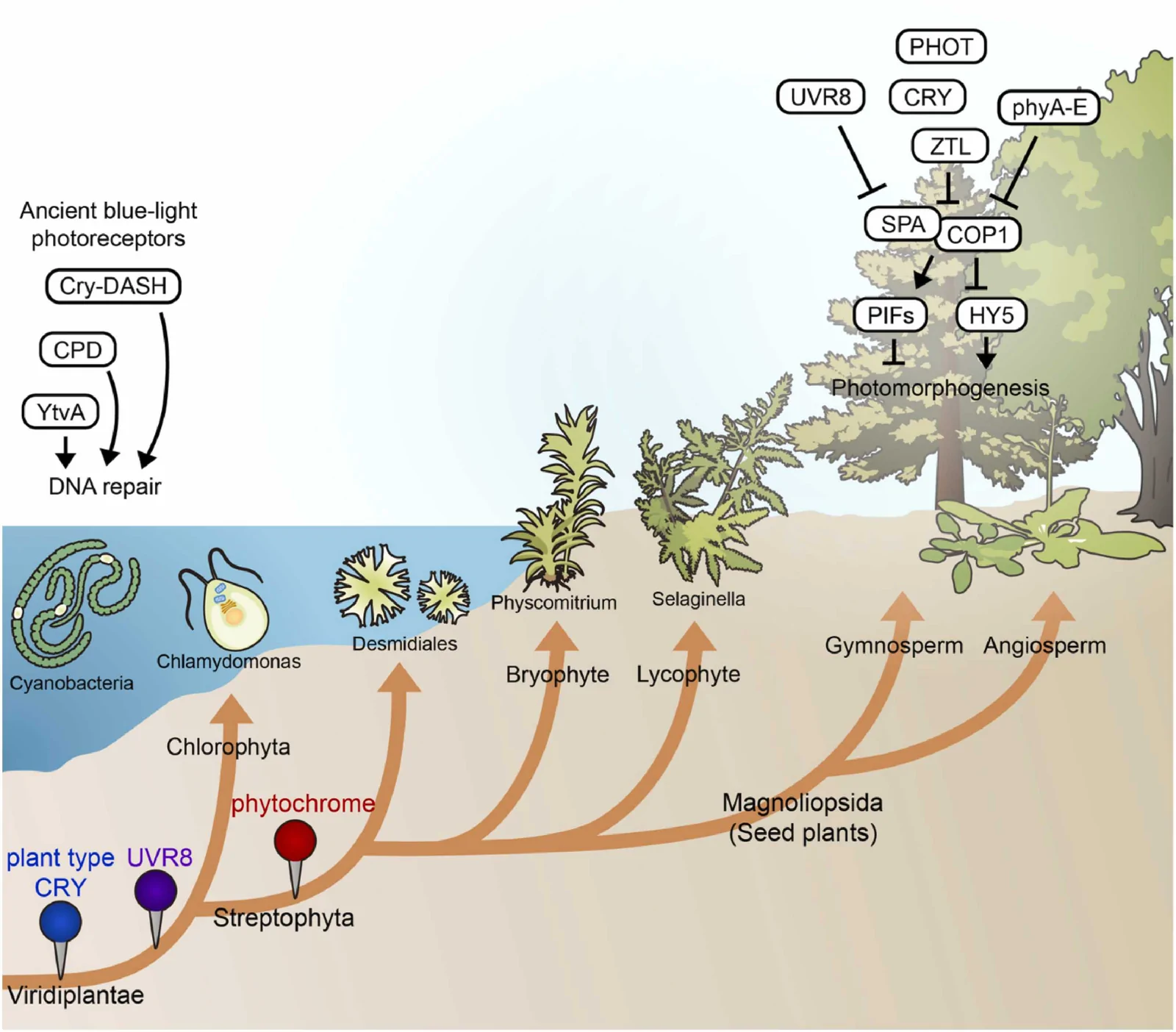
*Evolutionary expansion of light perception and signaling pathways in photosynthetic organisms.* The progressive diversification of photoreceptor systems and light-responsive signaling networks from green algae to land plants is illustrated. In ancestral chlorophyte lineages, blue light photoreceptors, CRY, PHOT, and other LOV-domain proteins, together with the UV-B photoreceptor, primarily mediated rapid physiological responses. As plants colonized land, bryophytes and early vascular plants acquired an expanded repertoire of photoreceptors, including multiple phytochromes, enabling spectral discrimination and transcriptional regulation of photomorphogenic processes. In seed plants, with the framework most extensively characterized in angiosperms, light signaling networks became hierarchically organized around conserved transcriptional modules. These include photoreceptor-dependent regulation of the E3 ubiquitin ligase COP1 and its associated factor, SPA, leading to coordinated control of PIFs and HY5.

Light perception and response have been defining characteristics of photosynthetic organisms since the earliest stages of evolution. From unicellular algae to land plants, photoreceptors have provided a molecular interface between environmental light cues and cellular behavior for their development and survival (Fig. 1). In ancestral aquatic lineages such as *Chlamydomonas reinhardtii* and other green algae, photoreceptors primarily functioned to synchronize light-directed movement (phototaxis), chloroplast movement, and circadian rhythms with diurnal cycles. These functions relied on ancient blue-light photoreceptors—cryptochromes and phototropin-like LOV-domain proteins—that directly coupled photon absorption to changes in redox state, calcium signaling, or second messengers, without the need for complex transcriptional cascades (Han et al., 2023b, Kianianmomeni and Hallmann, 2014, Kottke et al., 2017). In bryophytes such as *Physcomitrium patens*, the photoreceptor repertoire expanded to include multiple phytochromes and cryptochromes, allowing spectral discrimination and enabling the transcriptional regulation of photomorphogenic processes (Li et al., 2015, Mei and Dvornyk, 2015, Suetsugu and Wada, 2013). These early layers established the foundation for the hierarchical signaling networks later elaborated in photomorphogenic responses of vascular plants (Han et al., 2019, Huq et al., 2024). In land plants, light is perceived by multiple classes of photoreceptors that respond to distinct regions of the light spectrum, including phytochromes for red and far-red light, cryptochromes, phototropins, and Zeitlupe family proteins for blue and UV-A light, and UVR8 for UV-B light. Through these wavelength-specific receptors, light regulates a broad range of canonical developmental and physiological processes, including seed germination, phototropism, chloroplast movement, stomatal responses, shade avoidance, circadian entrainment, flowering, and senescence (Casal, 2013, Cerdán and Chory, 2003, Galvão and Fankhauser, 2015, Sanchez et al., 2020). These functions are mediated by well-characterized regulatory mechanisms such as COP1/SPA-dependent protein turnover, PIF regulation, HY5-mediated transcriptional control, and photoreceptor-specific kinase or ubiquitin ligase activities, which have been extensively summarized in recent comprehensive reviews (Balcerowicz, 2020, Gangappa and Botto, 2016, Leivar et al., 2008, Xiao et al., 2022). These canonical frameworks define much of the molecular logic of light signaling, yet they represent only one subset of the photoreceptor’s broader biological repertoire.

Evolutionary diversification of photoreceptors has been accompanied by the acquisition of new regulatory capacities, enabling integration with hormonal, redox, and metabolic pathways that mediate physiological and developmental plasticity. As a result, photoreceptors now serve as molecular integrators that translate not only light but also non-light environmental cues into developmental outcomes across tissues and life stages. Recent findings begin to reveal that photoreceptor-related signaling can operate in organs or conditions where light is minimal or absent—such as roots, vascular tissues, and stress-challenged cells. In these contexts, photoreceptors may act through direct protein–protein interactions, post-translational regulation, and hormonal crosstalk, in some cases independently of direct photoperception. This conceptual shift—from photoreceptors as “light sensors” to “environmental integrators”—reflects an emerging view that light signaling modules have been co-opted throughout plant evolution to control developmental plasticity and stress adaptation under fluctuating environments.

In this review, we use the term “non-canonical photoreceptor functions” broadly to describe photoreceptor-mediated roles that extend beyond the classical seedling-centered framework of photomorphogenesis, shade avoidance, and circadian entrainment. This term is intended to define the scope of the review rather than a single mechanistic class. Within this scope, tissue- or context-specific functions refer to photoreceptor activities in organs or developmental settings that have been less emphasized in classical light signaling studies, such as roots and vascular tissues, which may still involve local, transmitted, or systemic light-derived signals. By contrast, light-independent functions refer more specifically to cases in which photoreceptors exert regulatory effects or undergo stabilization or activation in the absence of direct light exposure.

Here, we summarize recent discoveries that expand the scope of photoreceptor functions beyond photomorphogenesis. We discuss how photoreceptors integrate light, temperature, hormonal, and redox cues to regulate abiotic stress responses; how they function in subterranean and vascular tissues to coordinate local and systemic signaling; and how these mechanisms shape seasonal growth and adaptation in woody plants. By tracing the evolutionary and mechanistic diversification of photoreceptor signaling, we aim to provide a perspective on how plants repurpose light-sensing modules to orchestrate developmental and physiological flexibility—an essential trait for survival and productivity across ecological and evolutionary timescales.

### Photoreceptors in Abiotic Stress Responses

Photoreceptors, traditionally recognized for their role in light-mediated development, have increasingly been identified as versatile regulators of plant abiotic stress. Beyond canonical photoperception, these sensors coordinate responses to drought, salinity, and oxidative stress through direct protein-protein interactions, hormonal crosstalk, and long-distance signaling. Notably, many of these functions occur through non-canonical or even light-independent mechanisms, positioning photoreceptors as central integrators of environmental cues.

**Fig. 2:**
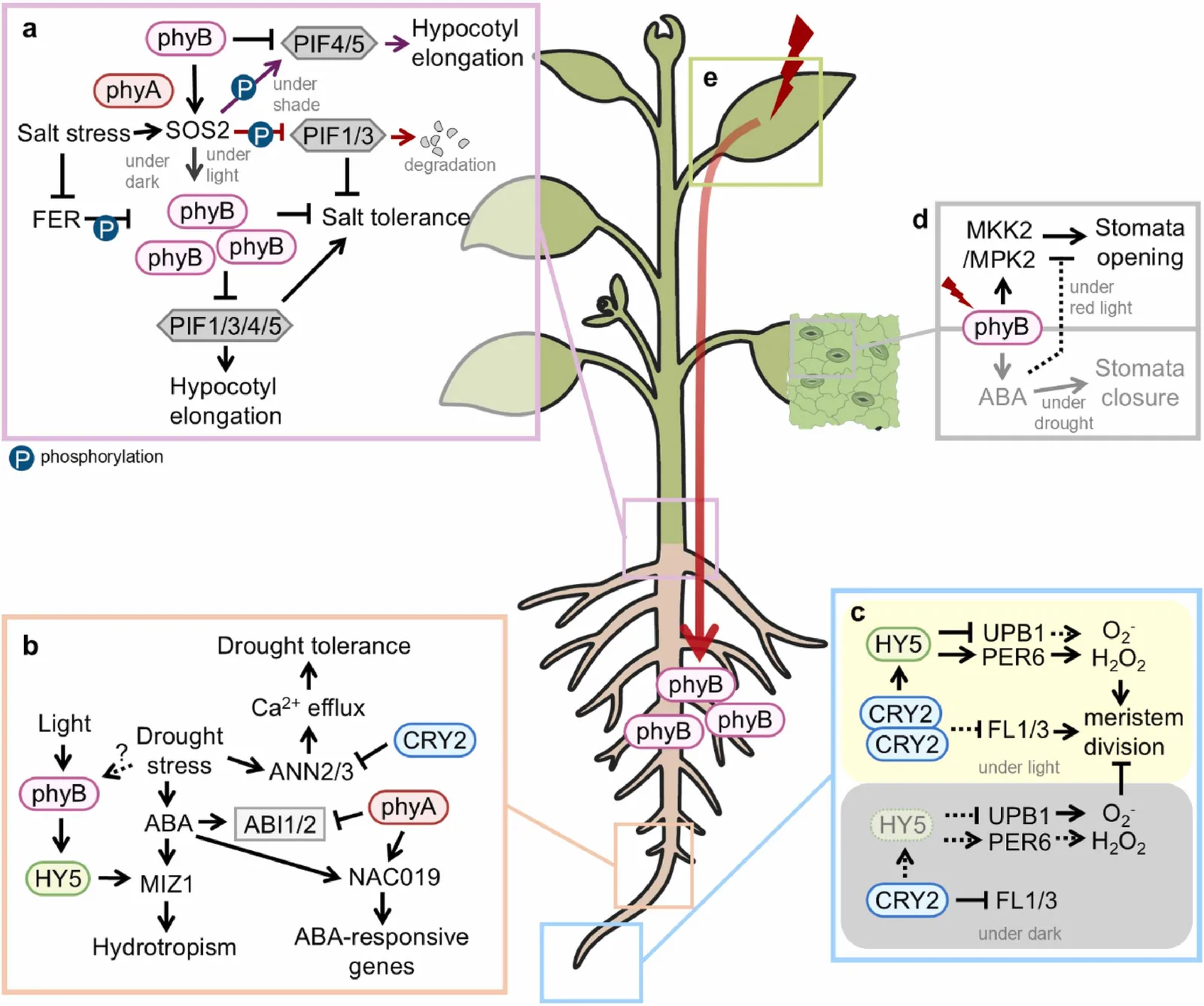
*Function of photoreceptors in abiotic stress responses and light-independent action in root meristem development.***a**, Salt stress connects SOS2, phyB, and PIF signaling. Salt-activated SOS2 promotes phyB photobody formation in darkness and enhances PIF1/3 degradation. Under light, phyA and phyB further increase SOS2 kinase activity, leading to SOS2-mediated phosphorylation and degradation of PIF1/3 and enhanced salt tolerance. Under shade, SOS2 phosphorylates and stabilizes PIF4/5 by reducing their interaction with phyB, thereby promoting shade avoidance. Salt stress also inhibits FERONIA (FER), which normally limits phyB photobody formation. **b**, Crosstalk between drought/ABA signaling and photoreceptors. phyB–HY5 induces MIZ1 expression during root hydrotropism, CRY2 attenuates ANN2/ANN3-mediated calcium signaling, and phyA regulates ABA responses through interactions with ABI1/ABI2 and transcriptional control of NAC019. **c**, Photoreceptor-mediated regulation of root meristem activity. In darkness, monomeric CRY2 inhibits FL1/FL3 to reduce cell division, whereas light-induced CRY2 oligomerization releases this inhibition. HY5 also promotes meristem activity by regulating PER6 and UPB1 expression and modulating ROS balance. **d**, Dual roles of phyB in stomatal regulation. phyB promotes stomatal opening under red light through the MKK2–MPK2 cascade but enhances ABA-mediated stomatal closure during drought. ABA-activated SnRKs further suppress phyB-mediated stomatal opening. **e**, Stem-piped light signaling. Red light perceived in aerial tissues can be transmitted to roots, where it activates phyB in otherwise dark-grown root tissues.

Recent studies have highlighted that photoreceptors modulate drought and osmotic stress adaptation by regulating hormone sensitivity, reactive oxygen species (ROS) detoxification, and stress-responsive signaling in roots (Fig. 2b). In rice, phytochrome B (phyB) acts as a negative regulator of drought tolerance by repressing ROS-scavenging enzymes and promoting leaf expansion, whereas *osphyB* mutants show enhanced drought resistance with improved ROS detoxification and reduced stomatal density (Yoo et al., 2017). Similarly, in *Arabidopsis*, CRY2 suppresses drought adaptation by physically interacting with annexins AtANN2 and AtANN3, which mediate Ca²⁺ influx under osmotic stress. CRY2 prevents their membrane localization, thereby attenuating stress signaling in a photoperiod-dependent manner, even in root tissues with minimal light exposure (Liu et al., 2021). Beyond ROS homeostasis, photoreceptors also modulate hormonal signaling pathways that coordinate stress adaptation, especially through interactions with abscisic acid (ABA) (Mahapatra et al., 2025). Under well-watered conditions, red light promotes stomatal opening via phyB, which activates a phosphorylation cascade involving MITOGEN-ACTIVATED PROTEIN KINASE KINASE 2 (MKK2) and MITOGEN-ACTIVATED PROTEIN KINASE (MPK2) (Li et al., 2023b, Wang et al., 2010). Under drought, however, phyB enhances ABA responsiveness and promotes stomatal closure, thereby contributing to drought tolerance (Gonzalez et al., 2012). Conversely, SUCROSE NON-FERMENTING-1 KINASEs (SnRKs), key kinases that activate ABA signaling, directly interact with and inhibit phyB to suppress red light-induced stomatal opening (Li et al., 2021b). Light and ABA signals also converge on MIZU-KUSSEI1 (MIZ1), a gene essential for root hydrotropism and known to be induced by ABA. *MIZ1* expression is upregulated by phyA, phyB, and HY5, integrating light perception with water-sensing in roots. Mutants lacking *MIZ1* or these photoreceptors exhibit impaired hydrotropic curvature, highlighting the relevance of light-regulated signaling in below-ground drought adaptation (Moriwaki et al., 2012).

Further evidence for hormone-photoreceptor integration arises from direct molecular interactions with ABA signaling components. Light-activated phytochrome A (phyA) physically interacts with ABI1 and ABI2 phosphatases, disrupting their association with ABA receptors and reducing ABA sensitivity, a mechanism validated in yeast-based assays (Li et al., 2023a). Moreover, phyA directly binds to the *NAC019* promoter, modulating stress-responsive gene expression at the transcriptional level (Chen et al., 2014). Remarkably, some photoreceptor functions occur entirely independently of light. For example, in the *Arabidopsis* root meristem, phyA promotes genome stability by activating DNA repair and cell-cycle checkpoint genes under genotoxic stress—even in darkness (Huerta-Venegas et al., 2024).

A compelling aspect of photoreceptor function in abiotic stress is their post-translational regulation during salt stress (Park et al., 2016) (Fig. 2a). The receptor-like kinase FERONIA (FER) directly interacts with and phosphorylates phyB, modulating its nuclear abundance via photobody stability. Under saline conditions, FER activity is suppressed, resulting in phyB accumulation, which attenuates salt tolerance (Liu et al., 2023). Intriguingly, salt stress also induces phyB nuclear body formation in darkness, mediated by SALT OVERLY SENSITIVE2 (SOS2) kinase and 14–3–3κ proteins. Activated phyB suppresses PIF3 activity, thereby mediating the inhibitory effect of salt on hypocotyl elongation in darkness (Qi et al., 2025). These selected examples suggest that non-light stress cues can modulate phyB stability or subnuclear organization, thereby expanding the potential signaling contexts in which photoreceptors operate. Photoreceptors also interact with the SOS2–PIF signaling module to dynamically regulate stress and growth. Under salt stress, phyA and phyB enhance SOS2-mediated phosphorylation and degradation of PIF1 and PIF3, promoting salt tolerance (Ma et al., 2023). In contrast, under shaded light, SOS2 phosphorylation attenuates the interaction between phyB and PIF4/5, stabilizing PIF4/5 to promote elongation growth (Han et al., 2023a). This isoform-specific modulation of PIFs highlights the flexibility of photoreceptor signaling in balancing growth and stress adaptation under distinct environmental cues (Jeong & Choi, 2013) (Fig. 2a).

In addition to local responses, photoreceptors mediate systemic stress signaling across tissues. For instance, phyB initiates a ROS wave that propagates from light-stressed leaves to neighboring tissues, inducing systemic stomatal closure and acquired acclimation (Devireddy et al., 2020, Fichman et al., 2023). Moreover, phyB enhances the unfolded protein response during endoplasmic reticulum (ER) stress by upregulating ER-resident luminal binding protein 2/3 (BiP2/3) and bZIP transcription factors, linking light signaling to ER homeostasis (Ahn et al., 2022). Adding further complexity, retrograde signaling from plastids influences photoreceptor stability and function. The stress-induced metabolite, methylerythritol cyclodiphosphate, stabilizes phyB, enhancing red light signaling and coordinating with auxin and ethylene pathways to restrict hypocotyl elongation (Jiang et al., 2019). Together, these findings underscore that photoreceptors regulate abiotic stress responses not only through light perception but also via direct protein interactions, hormonal modulation, and systemic signaling—often functioning beyond canonical, light-dependent paradigms.

### Photoreceptors in Temperature Stress Responses

Photoreceptors are well established as regulators of ambient temperature sensing and thermomorphogenesis. In Arabidopsis, phyB functions as a thermosensor through temperature-dependent thermal reversion from the active Pfr state to the inactive Pr state, thereby linking light perception with warm-temperature growth responses (Jung et al., 2016, Legris et al., 2016). During the daytime, phyB-mediated thermosensing also involves PIF4 and the transcriptional activator HEMERA (HMR), which together regulate temperature-responsive growth-related genes (Qiu et al., 2019). In addition, UV-B perception by UVR8 can attenuate thermomorphogenesis by repressing PIF4 accumulation and activity (Hayes et al., 2017). However, because ambient temperature sensing and thermomorphogenic growth have been extensively reviewed elsewhere (Casal et al., 2024, Delker et al., 2022, Quint et al., 2023), this section focuses primarily on photoreceptor functions in cold, freezing, and heat stress responses.

Temperature stress represents a major environmental challenge for plants, affecting growth, development, and survival. Recent findings underscore that photoreceptors act as integrators of light and temperature cues, modulating hormonal, redox, metabolic, and transcriptional pathways to coordinate stress acclimation.

In response to cold stress, photoreceptors regulate hormonal pathways that promote acclimation. In tomato, far-red light–activated phyA induces ABA biosynthesis, which subsequently activates jasmonic acid signaling and upregulates C-REPEAT BINDING FACTOR (CBF)-mediated cold-responsive genes. Conversely, phyB, activated under red light, suppresses this pathway. Genetic analyses indicate that ABA acts upstream of jasmonic acid and that phyA plays an essential role in cold acclimation under far-red light (Wang et al., 2016).

In Arabidopsis, phytochrome signaling also contributes to cold-induced transcriptional activation of the CBF regulon. Kim et al showed that light enhances cold-induced expression of C/DRE-mediated reporter genes and endogenous cold-responsive genes, including CBF1, CBF2, CBF3, and COR15a. Analysis of phyA, phyB, and phyA phyB mutants further suggested that phyB plays a major role in mediating light-dependent cold-responsive gene expression through the C/DRE pathway (Kim et al., 2002). More recently, a mechanistic link between cold signaling and phyB activity was provided by the CBF–PIF3–phyB module. Under cold stress, CBFs interact with PIF3 and attenuate PIF3–phyB co-degradation, thereby stabilizing phyB. Stabilized phyB subsequently promotes the degradation of PIF1, PIF4, and PIF5, negative regulators of freezing tolerance, and enhances freezing tolerance by modulating stress-responsive and growth-related genes (Jiang et al., 2020).

Consistently, light quality and intensity modulate cold acclimation and freezing tolerance in Arabidopsis. Photoreceptor mutant analyses showed that phyA, phyB, CRY1, and CRY2 contribute to freezing tolerance, with CRY1 being particularly important under optimal light intensity and phyA under low-light conditions (Prerostova et al., 2021).

Cold stress adaptation can also involve light-dependent photoreceptor signaling beyond direct transcriptional regulation. In Arabidopsis, freezing treatment under light enhances tolerance to subsequent freezing exposure. This response requires phototropins (PHOT1/PHOT2), phyB, and active photosynthetic electron transport, suggesting that photoreceptors may contribute to cold acclimation by linking light perception with photosynthetic energy metabolism and ROS production, in addition to transcriptional cold-response pathways (Sugita et al., 2024).

Because elevated ambient temperature and heat stress are sometimes described using overlapping terminology, temperature conditions should be specified when discussing heat-related responses. For example, Song et al examined Arabidopsis responses to defined 37°C treatments, which the authors referred to as heat stress. A short-term 37°C treatment for 30 min induced heat-responsive transcriptomic changes, including genes associated with light signaling and phytohormone-related pathways. Moreover, under prolonged exposure to 37°C, phyB mutants exhibited higher survival and reduced lateral root inhibition compared with wild-type plants, suggesting that phyB modulates plant responses to elevated temperature in a light-dependent manner (Song et al., 2017).

In another high-temperature context, phyB regulates seed germination through redox-sensitive signaling. Under high ambient temperature (32°C), phyB promotes germination by stabilizing the transcription factor LONG HYPOCOTYL IN FAR-RED1 (HFR1), which is otherwise degraded via nitric oxide (NO)-mediated S-nitrosylation. phyB enhances the activity of S-nitrosoglutathione reductase (GSNOR), thereby reducing NO accumulation and preventing HFR1 degradation, supporting germination under thermal stress high ambient temperature conditions (Ying et al., 2022). This reveals a non-canonical role for phyB in controlling the stability of a redox-sensitive transcription factor during high-temperature germination.

Blue-light receptors also contribute to thermotolerance under severe heat-stress conditions through CRY1-dependent regulation of heat-shock transcriptional networks. Under lethal heat stress conditions (45°C), Gao et al reported that CRY1 promotes light-induced thermotolerance by physically interacting with HEAT SHOCK TRANSCRIPTION FACTOR A1d (HSFA1d) and facilitating its nuclear localization through IMPORTIN α1. This mechanism links blue-light perception to the activation of thermotolerance-associated transcriptional programs under severe heat stress (Gao et al., 2023). In addition, Liu et al showed that heat stress-regulated CRY1 phosphorylation is critical for thermotolerance through the CRY1–COP1–HY5 module. Under heat stress (44°C), reduced CRY1 phosphorylation weakens the CRY1–COP1 interaction, allowing COP1-mediated HY5 degradation. Because HY5 represses the transcription of specific heat-shock transcription factor genes, heat-induced reduction of HY5 levels relieves this repression and increases HSF expression, thereby promoting thermotolerance. These studies suggest that CRY1 integrates blue-light and heat-stress cues through distinct regulatory modules involving HSFA1d nuclear localization and COP1–HY5–HSF signaling (Gao et al., 2023, Liu et al., 2025).

Overall, these findings demonstrate that photoreceptors coordinate temperature stress responses through multiple regulatory layers, including hormonal signaling, redox control, photosynthetic energy metabolism, and heat-shock transcriptional networks. Thus, beyond their canonical roles in light perception, photoreceptors serve as key environmental integrators that help plants adjust growth and survival strategies under fluctuating temperature conditions.

### Light-Independent and Root-Localized Functions of Photoreceptors in Root Development

Although roots are typically shielded from light, photoreceptors are widely expressed in root tissues and contribute to developmental and stress-responsive processes. While many photoreceptor functions in roots involve local light perception, recent findings suggest that photoreceptors can also operate independently of light, highlighting their non-canonical signaling roles in subterranean environments.

A compelling example of such light-independent function comes from a study on CRY2-mediated repression of root growth in darkness. In *Arabidopsis*, monomeric CRY2 binds to transcriptional regulators FORKED-LIKE 1 (FL1) and FL3, which promote root meristem cell division. In darkness, monomeric CRY2 inhibits FL1/FL3 activity, thereby reducing root growth. Upon blue light exposure, CRY2 oligomerizes, releasing FL1/FL3 and restoring meristem activity. This light-dependent switch functions as a developmental checkpoint controlled by the oligomerization state of CRY2. Importantly, the finding that CRY2 represses root growth even in complete darkness indicates that photoreceptors can exert regulatory activity in the absence of light, although whether additional photoreceptors share this capacity remains to be clarified (Zeng et al., 2025) (Fig. 2c).

In contrast, several studies have shown that roots can directly perceive light to regulate development through root-local photoreceptor activation. For example, HY5 is locally induced by light in root tissues, where it promotes the expression of *PEROXIDASE6 (PER6)* and represses *UPBEAT1 (UPB1)*, maintaining ROS balance and sustaining root meristem activity (Li et al., 2024). Notably, this response is independent of shoot light perception, demonstrating that roots can autonomously control stress signaling through localized photoreceptor activity. Similarly, RECEPTORS FOR ACTIVATED C KINASE 1A (RACK1A), a scaffold protein involved in brassinosteroid signaling, directly interacts with phyB and SUPPRESSOR OF PHYA-105 1 (SPA1) in roots to regulate meristem growth. While RACK1A stabilizes phyB–SPA1 interactions and promotes root development, its accumulation is enhanced by local light exposure, indicating that light-enhanced RACK1A activity contributes to early root meristem development (Zhu et al., 2024). This function aligns with observations that light transmitted through soil can influence root growth.

In addition to primary root regulation, photoreceptors also modulate root system architecture through local interactions with hormonal regulators. For example, phyB suppresses hypocotyl adventitious root formation by directly interacting with and stabilizing INDOLE-3-ACETIC ACID INDUCIBLE 14 (IAA14), thereby inhibiting AUXIN RESPONSE FACTOR 7/19 (ARF7/19)-mediated auxin signaling. This process occurs locally in hypocotyl tissues and demonstrates how photoreceptors can directly regulate organogenesis through hormone signaling components, independent of systemic cues (Li et al., 2021a). Further support for root-local photoreceptor activity is provided in the review by Warnasooriya & Montgomery, 2011, which summarizes evidence that phyA and phyB in *Arabidopsis* roots regulate root elongation, root hair formation, and gravitropic responses. Collectively, these findings emphasize the spatial specificity of photoreceptor function and reinforce the concept that roots can autonomously perceive and respond to light to shape their own development (Warnasooriya & Montgomery, 2011).

Despite these advances, CRY2 remains the only photoreceptor with a clearly demonstrated light-independent role in root development. However, other photoreceptors, such as phyB, have been shown to be activated by non-light environmental stimuli. For instance, salt stress induces phyB nuclear body formation even in darkness, suggesting that certain stress cues can alter phyB subnuclear organization independently of light (Qi et al., 2025). These observations raise the possibility that light-independent photoreceptor signaling is more prevalent than currently appreciated, particularly in subterranean tissues naturally shielded from light.

The examples discussed above highlight root-local photoreceptor activity, in which photoreceptors or light-responsive factors act within root tissues. In addition to these local mechanisms, root development can also be influenced by light information perceived in aerial organs. Such shoot-to-root regulation may occur through direct light transmission to roots, as in stem-piped light, or indirectly through shoot-derived hormonal and metabolic signals.

Systemic regulation is exemplified by studies showing that light perceived in aerial organs can generate mobile signals that travel to the root, thereby modulating root growth and stress responses. For instance, shoot-derived blue light perception by cryptochromes promotes *DWARF4 (DWF4)* expression and brassinosteroid biosynthesis in root tips, enhancing root elongation (Sakaguchi et al., 2019). Similarly, phytochromes activated by shoot light or stem-transmitted light regulate root auxin homeostasis by repressing *YUCCA4/6 (YUC4/6)* expression through PIF inhibition (Spaninks & Offringa, 2023). Notably, stem-piped light can activate phyB photobody formation and induce light-responsive gene expression in roots, providing a direct route for aerial light perception to influence subterranean tissues (Lee et al., 2016).

Together, both local and systemic photoreceptor activities converge to regulate root plasticity, demonstrating that photoreceptors serve as central hubs for integrating environmental and endogenous cues to orchestrate root development, even in tissues with minimal light exposure.

### Photoreceptor-Mediated Control of Arabidopsis Vascular Tissues

Plant vasculature is generated by specialized stem cell niches in apical meristems and the vascular cambium to produce vascular tissues, including xylem and phloem. These tissues provide long-distance transport of water and nutrients, mechanical support, and plasticity in growth, establishing vasculature as a central hub for endogenous and environmental cues (Hunziker and Greb, 2024, Turley and Etchells, 2022). While the intrinsic molecular mechanisms governing vascular tissue development have been extensively studied (De Rybel et al., 2016, Miyashima et al., 2019, Shi et al., 2019, Smetana et al., 2019), the regulatory influence of light and its underlying molecular pathways has only recently begun to be elucidated.

**Fig. 3:**
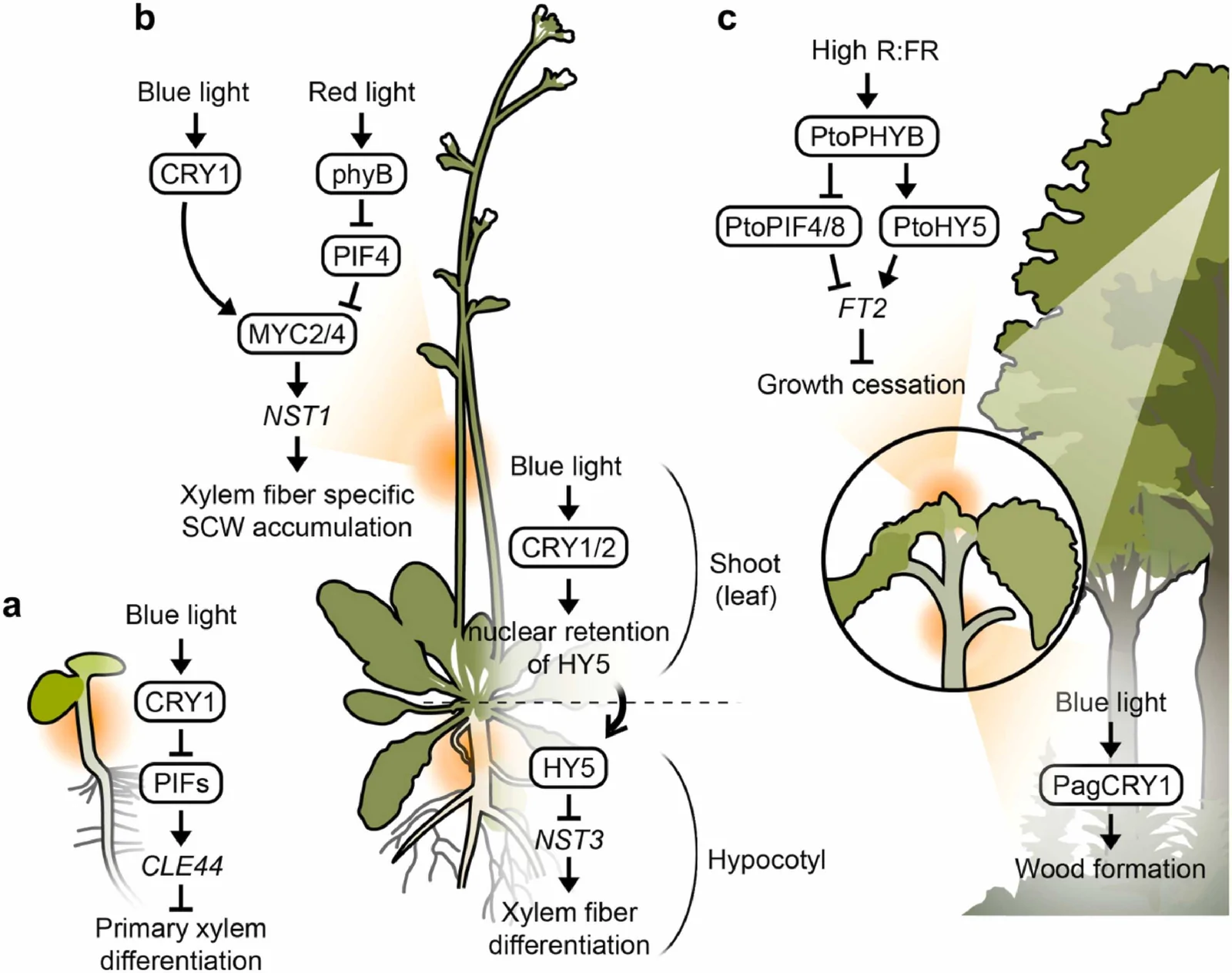
*Stage- and tissue-specific roles of light signaling in vascular development from primary growth to secondary growth.* This schematic summarizes light-mediated regulation of vascular development in Arabidopsis and woody plants. **a**, In dark-grown seedlings, PIF-dependent *CLE44* expression maintains procambial proliferation and suppresses primary xylem differentiation, whereas blue light-activated CRY1 inhibits PIFs to promote xylem differentiation. **b**, During Arabidopsis secondary growth, blue light regulates xylem fiber formation through distinct tissue-specific mechanisms, CRY1-MYC2/4-NST1 signaling in inflorescence stems and shoot CRY-dependent control of HY5 mobility and *NST3* expression in adult hypocotyls. **c**, In woody plants, phyB-PIF-FT modules regulate photoperiod-dependent growth cessation and dormancy, while CRY1 promotes libriform fiber differentiation and SCW accumulation.

Time-series single-cell transcriptomics revealed that light induces highly heterogeneous transcriptional responses across shoot tissues, with mesophyll and epidermal cells showing extensive reprogramming, whereas vascular tissues remain transcriptionally stable. Despite this apparent stability, light responses within the vasculature are not uniform but instead exhibit cell-type-specific patterns. In particular, procambial cells act as key nodes where light perception redirects developmental trajectories, inducing vascular development toward phloem differentiation while suppressing xylem development (Han et al., 2023b). This suggests that such selective light responsiveness supports the establishment of functional phloem connectivity required for efficient transport of photosynthates. The balance between cell division and differentiation drives the formation of plant vascular tissues. While procambium-derived cells differentiate into xylem and phloem lineages, seedlings germinated in darkness exhibit reduced xylem differentiation, with hypocotyl metaxylem remaining discontinuous and cotyledon venation restricted to the primary vein. In contrast, light exposure promotes the development of xylem cells, suggesting that light is essential for establishing vascular cell differentiation identity during primary growth. Within this framework, CLE44 acts as a potent inhibitor of light-induced xylem differentiation. In darkness, PIF transcription factors accumulate and promote *CLE44* expression, which activates TDIF-PXY signaling to maintain procambial proliferation while suppressing xylem differentiation. Under blue light, CRY1 inactivates PIFs, leading to decreased *CLE44* expression and the release of xylem differentiation programs. Thus, light quality acts as a developmental switch that controls the balance between procambial proliferation and xylem differentiation (Ghosh et al., 2022) (Fig. 3a).

During the evolution of land plants, competition for light promoted the development of taller and thicker plant bodies supported by specialized vascular tissues (Tonn and Greb, 2017, Woudenberg et al., 2022). Blue-light/CRY signaling continues to shape vascular differentiation, but through distinct, tissue-specific mechanisms during secondary growth. In the inflorescence stem, CRY1 promotes secondary cell wall (SCW) thickening in xylem fiber cells by enhancing the activity of MYC2 and MYC4, which directly induce the master regulator NST1 and its downstream cellulose and lignin biosynthetic genes (Zhang et al., 2018) (Fig. 3b).

Whereas blue light-CRY1 signaling promotes SCW thickening in inflorescence stems through MYC2/4-mediated activation of NST1, CRY1 and CRY2 regulate xylem fiber formation in the adult hypocotyl via a distinct long-distance signaling mechanism involving HY5 (Hwang et al., 2024). Under blue light, CRYs retain HY5 in the nucleus of the shoot, preventing its translocation to the hypocotyl during secondary growth. Because HY5 represses the *NST3* promoter, a key regulator of SCW deposition in xylem fiber, retention of HY5 in shoot nuclei relieves this repression and enables xylem fiber differentiation. Moreover, blue light-CRY signaling upregulates SCW-related gene expression while concurrently repressing cell division-associated genes, suggesting that CRY signaling modulates the balance between cell division and differentiation during secondary growth (Fig. 3b).

Light quality further modulates the balance between stem elongation and SCW reinforcement through interactions between phyB, PIFs, and MYC transcription factors (Luo et al., 2022). Under high red/far-red (R:FR) light, active phyB destabilizes PIFs, allowing MYC2/4-driven *NST1* expression to promote SCW thickening, whereas low R:FR conditions permit PIF4 accumulation, which antagonizes MYC2/4 activity and suppresses SCW deposition. Together with blue light-CRY signaling, these findings indicate that photoreceptors regulate xylem fiber differentiation through organ- and context-specific transcriptional modules, including the CRY1-MYC2/4-NST1 pathway in inflorescence stems and a long-distance CRY-HY5-NST3 pathway in the adult hypocotyl (Hwang et al., 2024, Zhang et al., 2018) (Fig. 3).

### Photoreceptor Control of Seasonal Growth in Trees

Blue light-dependent induction of xylem fiber formation observed in *Arabidopsis* aligns with recent evidence from Populus showing that CRY1-mediated signaling regulates wood development (Chen et al., 2024, Hwang et al., 2024). These findings point to a shared developmental strategy in which light quality information is incorporated into secondary growth programs (Fig. 3c).

In perennial woody plants, photoreceptor signaling operates not only at the level of cellular differentiation but also at the scale of seasonal growth regulation. Seasonal growth regulation encompasses coordinated transitions between active growth and dormancy, including growth cessation, bud dormancy, and subsequent reactivation, as well as pronounced changes in cambial activity and seasonal growth. Although photoperiod and temperature have long been recognized as key regulators of growth cessation and bud break in trees, recent studies have clarified how light and photoreceptor signaling mediate these seasonal transitions. In *Populus*, red/far-red light signaling through phyB-PIF modules coordinates seasonal growth by regulating FT homologs, thereby integrating photoperiodic cues with growth cessation and bud break (Ding et al., 2021, Zhang et al., 2025). Notably, phyB acts as a positive regulator of vegetative growth under permissive conditions, whereas PIFs function as negative regulators that promote growth cessation, revealing a photoreceptor-based switch controlling seasonal growth transitions (Zhang et al., 2025). In addition to phyB-PIF signaling, photoperiodic regulation of bud dormancy involves a HY5-centered network that integrates light perception with circadian and hormonal pathways. In *Populus tomentosa*, HY5 modulates *FT* expression and gibberellin homeostasis, thereby contributing to both dormancy entry and release (Gao et al., 2024). Beyond controlling seasonal growth transitions, photoreceptor signaling also extends its influence to the regulation of secondary growth and wood formation in woody plants. Blue-light signaling also plays a direct role in secondary growth, as CRY1 promotes xylem lignification and SCW accumulation while suppressing cambial cell division through HY5- and BBX-dependent transcriptional regulation in *Populus* (Chen et al., 2024). Together, these studies demonstrate that photoreceptor signaling integrates seasonal light cues with hormonal and developmental programs to coordinate growth-dormancy transitions and wood formation in perennial woody plants (Fig. 3).

## CONCLUSION AND PERSPECTIVE

Plant photoreceptors, once viewed solely as light sensors orchestrating photomorphogenesis, are now recognized as versatile integrators of diverse environmental signals. Beyond their canonical roles, photoreceptors regulate abiotic stress adaptation, root and vascular development through non-canonical pathways, including direct protein interactions, post-translational regulation, and hormone signaling (Ghosh et al., 2022, Hwang et al., 2024, Lau and Deng, 2010). Notably, recent findings demonstrate that photoreceptors can function in darkness or in tissues with minimal light exposure, highlighting their ability to respond to non-light environmental cues and coordinate developmental plasticity. Taken together, these findings reposition photoreceptors as multifaceted regulators of adaptation.

This expanded repertoire may reflect the evolutionary refinement of pre-existing light-sensing modules. Although major plant photoreceptor and light-signaling mechanisms emerged stepwise as photosynthetic lineages adapted to changing light-quality gradients in aquatic and terrestrial environments, the increasing complexity of land plant habitats likely favored further functional specialization (Han et al., 2019). Following terrestrialization, environmental pressures such as direct sunlight, soil emergence, water limitation, temperature fluctuation, light competition, canopy shade, and seasonal changes may have promoted gene duplication, paralog diversification, and rewiring of downstream signaling networks (Zhang et al., 2022). Such evolutionary redeployment may have enabled photoreceptors to coordinate tradeoffs between growth, stress resilience, developmental progression, and energy allocation across diverse tissues and developmental stages.

Previous studies and recent reviews have established that light signaling components can integrate multiple environmental cues, including temperature, drought, salinity, and biotic stress (Iqbal et al., 2021, Ma et al., 2023; Mukherjee et al., 2023; Qi et al., 2022). However, less is known about how photoreceptor-specific modules, hormonal crosstalk, and downstream regulators are redeployed in less-explored developmental contexts, including roots, vascular tissues, and perennial woody growth. Likewise, while several examples indicate that photoreceptors or light signaling components can be modulated by non-light cues, the molecular principles that determine how plants balance growth, stress tolerance, and energy allocation across tissues and environmental conditions are not fully resolved. Further comparative studies across non-vascular plants, herbaceous angiosperms, and woody species will help clarify how these non-canonical photoreceptor functions have been conserved, modified, or diversified during plant evolution. Addressing these gaps will deepen our understanding of how plants exploit environmental information to fine-tune survival strategies.

By moving beyond the canonical paradigm of photomorphogenesis, we can now appreciate photoreceptors as central hubs of environmental signal integration. Their hidden, non-canonical roles—in vascular differentiation, stress adaptation, and root development—illustrate the remarkable plasticity of plant signaling systems. Future research aimed at disentangling these diverse functions will not only expand our conceptual framework of photoreceptor biology but also provide new opportunities to enhance plant resilience and productivity in the face of global environmental change.

## CRediT authorship contribution statement

**Hyeona Hwang:** Writing – review & editing, Writing – original draft. **Soeun Han:** Writing – review & editing, Writing – original draft, Supervision, Conceptualization. **Hyunwoo Cho:** Writing – review & editing, Writing – original draft, Supervision, Conceptualization.

## Declaration of Competing Interests

The authors declare that they have no known competing financial interests or personal relationships that could have appeared to influence the work reported in this paper.
